# Renal complications in coronavirus disease 2019: a systematic review

**DOI:** 10.1186/s41232-020-00140-9

**Published:** 2020-12-15

**Authors:** Taichiro Minami, Yasunori Iwata, Takashi Wada

**Affiliations:** 1 Renal Division, Department of Medicine, Brigham and Women’s Hospital, 4 Blackfan Street, Boston, MA USA; 2 Department of Nephrology and Laboratory Medicine, Kanazawa University, Kanazawa, Ishikawa Japan

**Keywords:** COVID-19, Renal complication, AKI

## Abstract

The world today is facing a pandemic caused by severe acute respiratory syndrome coronavirus-2 (SARS-CoV-2), which mainly causes a respiratory disease known as coronavirus disease 2019 (COVID-19). Therefore, its pathogenesis and complications should be identified and understood. SARS-CoV-2 infects the host using the angiotensin-converting enzyme 2 (ACE2) as its receptor, which is expressed in several organs including the lungs, heart, kidneys, and intestines. Kidney complications are relatively common, and acute kidney injury (AKI) is a life-threatening complication in patients with COVID-19. In this review, the renal histological patterns of COVID-19 are described in detail, and its potential mechanisms associated with AKI are discussed.

## Background

In the late 2019, a novel coronavirus (severe acute respiratory syndrome coronavirus 2 [SARS-CoV-2]) was identified as the cause of a cluster of pneumonia cases in Wuhan, China. This outbreak has led to a worldwide pandemic within a short period of time, and as of June 21, 2020, there are more than 8.7 million confirmed cases with 460,000 deaths in 216 countries, areas, or territories according to the World Health Organization.

Although lung infection and acute respiratory failure are the main characteristics of coronavirus disease 2019 (COVID-19), other organs such as the heart, small intestines, and kidneys are also reportedly involved.

Acute kidney injury (AKI) is defined as increased serum creatinine level (by 0.3 mg/dL within 48 h or > 1.5 times from its baseline level) or decreased urine output (< 0.5 mL/kg/h for 6 h) according to the Kidney Disease Improving Global Outcomes guideline. AKI is one of the important complications of COVID-19, occurring in 0.5–7% of cases and in 2.9–23% of intensive care unit (ICU) patients [1–3]. A single-center prospective cohort study of 701 inpatients with COVID-19 in China reported that 43.9% of patients had proteinuria and 26.7% had hematuria on admission and 5.1% developed AKI during hospitalization [4]. An Italian study involving > 2000 inpatients with COVID-19 reported the AKI incidence of 27.8% [5]. When compared with the AKI occurrence in severe acute respiratory syndrome (SARS), no significant difference was observed in the incidence, peak creatinine levels, and histological findings (Table 1).

**Table 1:** Comparison of clinical features and renal complication between SARS and COVID-19. Some data are presented as mean ± SD or median (IQR)

	SARS-CoV	SARS-CoV-2	Reference
Incubation time(days)	4.7(95%CI 4.3-5.1)	4.9(95%CI 4.5-5.5)	[6]
AKI incidence(%)	6.7	5.1	[4, 7]
AKI onset from admission(days)	7.2+4.3	4.0(2.0-7.5)	[8, 9]
Peak creatinine(mg/dL)	3.0(1.5-12.2)	2.2+2.4	[4, 7]
Autopsies findings	Acute tubular necrosis(7/7) Interstitial infiltration(0/7)	Acute tubular injury(26/26) Interstitial infiltration(non-specific)	[7, 10]
In hospital death(%)	14.3	12.5	[4, 7]

Understanding the COVID-19 pathogenesis is evolving. This review focuses on the potential AKI mechanism in COVID-19, especially the direct viral cytotoxicity to kidney host cells.

### Pathological features of kidney diseases in COVID-19

**Fig. 1:**
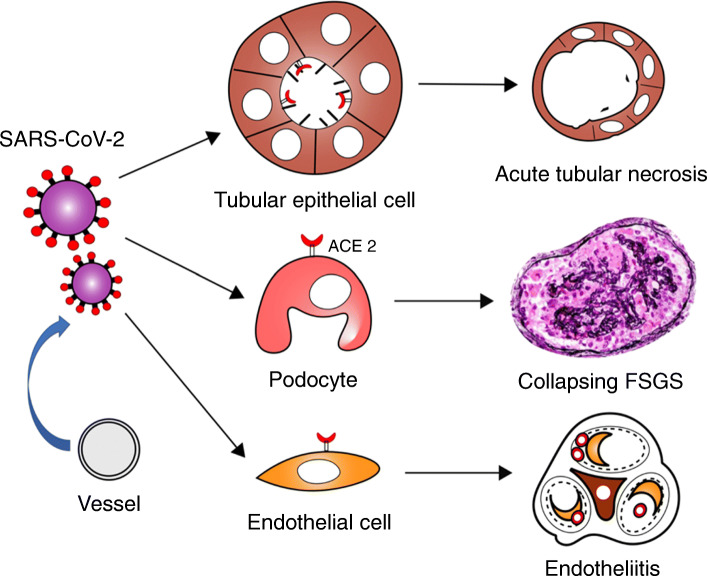
Schematic of histological features of renal complications in COVID-19. SARS-CoV-2 infects several kidney host cells using ACE2 and causes various types of damage. Each damage can cause AKI. Abbreviation: FSGS, Focal segmental glomerulosclerosis

Renal histopathological analysis of 26 autopsies with COVID-19 was reported in China. All specimens had acute tubular necrosis (ATN) findings, and 18 of these exhibited moderate to severe ATN. Diffuse proximal tubule injury was manifested as the loss of brush border, vacuolar degeneration, dilatation of the tubular lumen, detachment of epithelium, and even frank necrosis noted. Distal tubules and collecting ducts showed only occasional cellular swelling. Interstitial infiltrates were present but nonspecific and insignificant. They detected viral particles in tubular epithelial cells (TECs) and podocytes using a transmission electron microscope [10]. Post-mortem analysis of 42 patients from the USA also revealed ATN was the predominant finding and focal fibrin thrombi in 6 of 42 autopsies, whereas they could not identify the definitive virions at the ultrastructural level [11]. Although ATN is reported in many cases, whether viral infection directly leads to apoptosis in infected TECs remains unclear. Three separate case reports have described collapsing focal segmental glomerulosclerosis (FSGS) with nephrotic syndrome following a COVID-19 diagnosis in Africans (2 men and 1 woman). They developed rapid progressive renal function impairment, and 2 of 3 patients required dialysis. Kidney biopsies revealed ATN lesions and mild-moderate interstitial infiltrates in addition to glomerular lesion. No specific staining for IgG, IgA, IgM, C3, C1q, and kappa and lambda light chains in the glomeruli was found [12–14]. As virus-induced collapsed the FSGS, human immunodeficiency virus (HIV)-associated nephropathy is well studied. Transgenic mouse models indicate that certain HIV gene expressions such as Nef and Vpr are particularly involved in the pathogenesis of HIV-associated nephropathy. HIV-associated nephropathy is strongly associated with black race. SARS-CoV-2-related kidney damage may also occur based on endothelial cell injury. In histological analyses, endotheliitis of the heart, small intestine, and lung were described in patients with COVID-19 and characterized by inflammatory cell infiltrates within the intima and apoptosis of endothelial cells. Endotheliitis of glomerular capillaries was also reported in a post-mortem examination, and viral particle was confirmed in the endothelial cell [15]. These conditions may result in microvascular dysfunction leading to tissue inflammation, coagulopathy, and hypoxia. Based on kidney biopsy and autopsy findings, SARS-CoV-2 appears to infect certain kidney host cells and induce different types of damages (Fig. 1).

### AKI mechanisms in COVID-19

Early reports estimated AKI incidence in COVID-19 as 0.5–28% in China and Italy [1–5]. As a report from China described COVID-19 did not increase the AKI rate at all, AKI incidence appears to vary a lot [16]. This may occur from the population difference, disease severity, and AKI definition. In a recent report of over 5000 patients from the USA, AKI developed in 36.6% hospitalized patients with COVID-19. 37.3% of whom were diagnosed at the timing of admission or within 24 h after admission. Regarding AKI severity, stages 1, 2, and 3 were 46.5%, 22.4%, and 31.1%, respectively. They reported older age, diabetes mellitus, cardiovascular disease, black race, hypertension, and the need for ventilation and vasopressor medications as the risk factors for AKI in COVID-19 [17].

According to a prospective, multicenter study of 748 patients in Madrid, ATN and prerenal disease are the most common causes of AKI, comprising 45% and 21% of hospitalized patients, respectively [18]. Three major causes of ATN are renal ischemia, sepsis, and nephrotoxins.

Sepsis can also be considered as the cause of AKI in patients with COVID-19. A retrospective study of 191 patients with COVID-19 in Wuhan, China, revealed that 59% of patients had sepsis and 20% exhibited septic shock [19]. AKI is a type of multiple organ dysfunctions occurring in severe sepsis. Its pathophysiology during sepsis is complicated and multifactorial, including systemic hypotension, renal vasoconstriction, endothelial dysfunction, tubular cell damage, influx of inflammatory cells into the renal parenchyma, and capillary thrombosis. In addition, sepsis can activate the innate immune response and lead to a cytokine storm, most importantly in IL-1, IL-6, and TNF-α. A report of 452 patients with COVID-19 showed that serum levels of proinflammatory cytokines (TNF-α, IL-1, and IL-6) were found to be higher in the severe infection group than in the mild group. The median IL-6 levels in the severe group were 25.2 pg/mL (9.5–54.5; interquartile ratio [IQR]) and 13.3 pg/mL (3.9–41.1; IQR) in the non-severe group, with *p* value of < 0.001 [20]. In a mouse model study of sepsis-induced AKI, IL-6 depletion diminished neutrophil infiltration and caused resistance to renal injury [21]. Both TNF-soluble receptor and targeted deletion of TNF receptor 1 can prevent lipopolysaccharide-induced renal injury [22, 23]. Although endotoxin is thought to have an important role in the AKI occurrence in sepsis, blockade of proinflammatory cytokines may have a beneficial effect on AKI in patients with COVID-19 with robust cytokine storm [24].

Renal infarction in 2 patients with COVID-19 who both developed AKI without hemodynamic instability is described. Computed tomography images in both cases showed unilateral and multiple perfusion defects [25]. Coagulation disorders in patients with COVID-19 patients are suggested [26]. Patients with COVID-19 developed thrombocytopenia (36.2%), increased D-dimer (46.4%), and hypercoagulability, which was more serious in severely ill patients, according to a cohort study of 1099 patients from China [27]. Deep vein thrombosis (DVT) and pulmonary embolism (PE) commonly occur in ICU patients with COVID-19. A report of 150 ICU patients showed that 16.7% of the patients experienced PE and 2% had DVT, and 28 of 29 (96.6%) patients receiving continuous renal replacement therapies had clotting [28]. Regarding arterial thrombosis, central nervous system stroke and limb ischemia have been reported [29, 30]. Therefore, coagulation disorders and kidney infarction should be considered as the cause of AKI in patients with COVID-19.

**Fig. 2:**
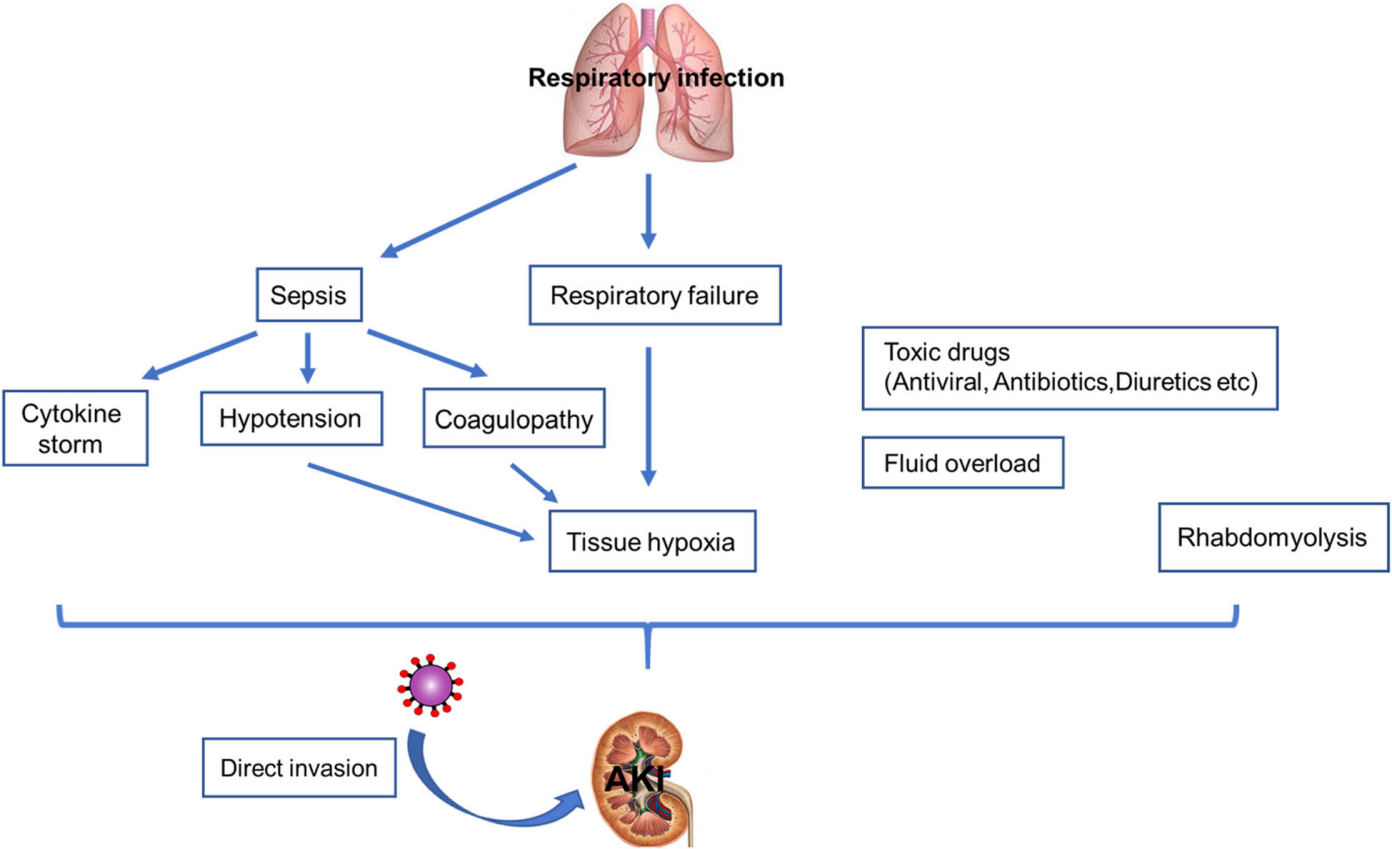
Possible AKI mechanisms in COVID-19. Etiologies of AKI are not fully understood. The causes appear to be multifactorial and wide-ranging, including direct viral invasion of kidney tissue, cytokine storm, hypotension, hypercoagulation, tissue hypoxia, toxic drugs, rhabdomyolysis

Respiratory failure is strongly related to AKI occurrence in COVID-19 according to an observational study. 89.7% of patients on mechanical ventilation developed AKI compared to 21.7% of non-ventilated patients [17]. A retrospective single-center study from China also described the severity of pneumonia was associated with the development of AKI [31]. Acute respiratory distress syndrome has been identified as a risk factor for AKI in critically ill patients [32]. Hypoxemia and hypoperfusion secondary to right heart failure may lead to ischemic kidney damage in COVID-19. Other AKI mechanisms such as toxic drugs and rhabdomyolysis are reported (Fig. 2) [33, 34].

### Mechanisms of SARS-CoV-2 direct invasion of host cells

SARS-CoV-2 has 79% genomic homology with SARS-CoV and belongs to the β coronavirus family. SARS-CoV sneaks into the host cells through an ACE2 receptor [35]. The spike (S) protein of the virus binds to ACE2, which then activated and cleaved by cellular transmembrane serine protease 2 (TMPRSS2). Thereafter, the virus releases fusion peptide for entry. In an in vitro study, SAR-CoV-2 was confirmed to use ACE2 for cellular entry and TMPRSS2 for S protein priming. Moreover, the TMPRSS2 inhibitor blocked the viral entry and antibodies from convalescent SARS patients’ sera also inhibited the invasion [36].

Since ATN is the most common finding in the kidney tissue from autopsies, SARS-CoV-2 may directly infect the hosts’ TECs. In a normal kidney, ACE2 is expressed in TECs as well as podocytes, vascular smooth muscle, and interlobular artery endothelium. Once the kidney is damaged, ACE2 is newly expressed in the glomerular endothelium and peritubular capillary [37]. The virus particles in TECs have been identified in patients with COVID-19 virtually [10]. However, whether SARS-CoV-2 impairs the TEC functions remains to be elucidated in further studies. ACE2 and TMPRSS genes are co-expressed in TECs and podocytes [38]. The virus particles in podocytes and endothelium are also shown [10, 15].

As the SARS-CoV-2 initially affects the upper respiratory tract, it needs to get into the bloodstream to reach the kidney. The SARS-CoV-2 RNAaemia is reported in 10–15% of patients with COVID-19 and particularly detected in critically ill patients [1, 39]. Furthermore, the rate of SARS-CoV-2 presence in the urine is 3.7% according to a meta-analysis review [40]. Thus, SARS-CoV-2 is considered to directly invade the kidney host cells through the bloodstream.

### The viral evasion system from the host immune response

In the kidney tissues, CD4+ T cells and CD56+ natural killer cells were hardly found in the kidney interstitium; however, severe infiltration of CD68+ macrophages (6/6 cases) and moderate CD8+ T cells (2/6 cases) were observed in six post-mortem examinations of patients with COVID-19 [41]. No lymphocyte infiltration was detected in the renal interstitium in an autopsy analysis of 7 patients with SARS in Hong Kong [7]. Consistent with these findings, infiltrated inflammatory cells in the alveoli were mainly macrophages and monocytes, and lymphocyte, neutrophil, and eosinophil infiltrates were found to be limited in a pathological report of three COVID-19 autopsies of lung tissues [42]. Another paper described focal lymphocyte infiltration accompanied by diffuse alveolar damage, the most common characteristic of COVID-19 pneumonia [43].

Some evidences also demonstrated that SARS-CoV-2 dysregulates T lymphocytes. SARS-CoV-2 directly infects T lymphocytes through the S protein-mediated membrane fusion and a peptide, which inhibits viral S protein, blocking the viral entry according to an in vitro study [44]. SARS-CoV envelope E protein has also been reported to bind to Bcl-xL and induce T cell apoptosis [45]. Other mechanisms such as destroying lymphatic organs and lymphocyte apoptosis induced by inflammatory cytokine storm have also been suggested [46]. Clinical data revealed that lymphopenia was found in 40% of patients with COVID-19 [47]. A retrospective study showed that low lymphocyte percentage was a predictor of COVID-19 severity. Decreased lymphocyte count over time was associated with poor outcome [46]. Another study reported that decreased CD4+ T cells were more evident in the peripheral blood of patients with severe COVID-19 [20]. Functional exhaustion of NK and CD8+ T cells is shown with increased NKG2A levels in patients with COVID-19 [47]. As these viruses can decrease the lymphocyte count and cause T cell dysfunction, lymphocyte infiltration in the renal interstitium may be limited considering the number of tubular necrosis.

## Conclusion

Based on kidney biopsy and autopsy findings, ATN is the most common lesion and can be caused by direct SARS-CoV-2 infection on TECs. Collapsing FSGS and endotheliitis are also induced by SARS-CoV-2 infection. Interstitial lymphocyte infiltration seems to be relatively suppressed and may be due to the viral evasion from the host immune system. Other potential mechanisms of AKI include renal ischemia, cytokine storm, toxic drug, thrombotic complication, and rhabdomyolysis. In-hospital AKI is associated with high mortality, and AKI severity is correlated with death in an AKI stage-dependent manner. Hence, early recognition of AKI and providing appropriate interventions are important to avoid further ATN and may be helpful to improve the prognosis of patients with COVID-19.

## Data Availability

Not applicable.

## Abbreviations

ACE2: Angiotensin-converting enzyme 2
AKI: Acute kidney injury
ATN: Acute tubular necrosis
COVID-19: Coronavirus disease 2019
FSGS: Focal segmental glomerulosclerosis
SARS: Severe acute respiratory syndrome
SARS-CoV: Severe acute respiratory syndrome coronavirus
SARS-CoV-2: Severe acute respiratory syndrome coronavirus 2
TECs: Tubular epithelial cells
TMPRSS2: Transmembrane serine protease 2
